# Comparison of Three Low-Molecular-Weight Fluorescent Probes for Measuring Free Zinc Levels in Cultured Mammary Cells

**DOI:** 10.3390/nu15081873

**Published:** 2023-04-13

**Authors:** Christopher Hübner, Claudia Keil, Anton Jürgensen, Lars Barthel, Hajo Haase

**Affiliations:** 1 Department of Food Chemistry and Toxicology, Institute of Food Technology and Food Chemistry, Technische Universität Berlin, Straße des 17. Juni 135, 10623 Berlin, Germany; 2 Department of Applied and Molecular Microbiology, Institute of Biotechnology, Technische Universität Berlin, Straße des 17. Juni 135, 10623 Berlin, Germany

**Keywords:** free zinc, ZinPyr-1, TSQ, FluoZin-3, breast cancer, MCF7, T47D, MDA-MB-231, MCF10A

## Abstract

Free zinc is a critical regulator in signal transduction and affects many cellular processes relevant to cancer, including proliferation and cell death. Acting as a second messenger, altered free intracellular zinc has fundamental effects on regulating enzymes such as phosphatases and caspases. Therefore, the determination of free intracellular zinc levels is essential to assess its influence on the signaling processes involved in cancer development and progression. In this study, we compare three low-molecular-weight fluorescent probes, ZinPyr-1, TSQ, and FluoZin-3, for measuring free zinc in different mammary cell lines (MCF10A, MCF7, T47D, and MDA-MB-231). In summary, ZinPyr-1 is the most suitable probe for free Zn quantification. It responds well to calibration based on minimal fluorescence in the presence of the chelator TPEN (*N*,*N*,*N*′,*N*′-Tetrakis(2-pyridylmethyl)ethylenediamine) and maximal fluorescence by saturation with ZnSO_4_, resulting in the detection of free intracellular zinc in breast cancer subtypes ranging from 0.62 nM to 1.25 nM. It also allows for measuring the zinc fluxes resulting from incubation with extracellular zinc, showing differences in the zinc uptake between the non-malignant MCF10A cell line and the other cell lines. Finally, ZinPyr-1 enables the monitoring of sub-cellular distributions by fluorescence microscopy. Altogether, these properties provide a basis for the further exploration of free zinc in order to realize its full potential as a possible biomarker or even therapeutic target in breast cancer.

## 1. Introduction

Globally, breast cancer is the most common cancer in women with regard to incidence and mortality [1]. The high degree of heterogeneity between the different subtypes of breast cancer in terms of molecular or histological characteristics provides a challenge for therapy. Optimal treatment requires early subtype characterization. Therefore, new potential biomarkers for breast cancer and their specific subtypes are coming into focus, and zinc is discussed as a potential biomarker for subtype specification in breast cancer [2,3]. It is known that zinc accumulates in breast cancer compared to healthy tissue or cell lines [4,5,6]. In addition, differences in the cellular and tissue amounts and the distribution of zinc were noted between the subtypes, making the link between zinc and breast cancer a promising approach for research [5].

In addition to the diagnostic potential, there might also be a functional role for zinc in breast cancer cells. Zinc is an essential trace element and a predicted co-factor of roughly 3000 proteins in humans, including approximately 300 enzymes [7]. Thus, zinc is involved in a myriad of cellular functions, including gene expression, cell death, and proliferation, all of which are highly relevant for cancer [8]. The vast majority of cellular zinc is tightly bound to proteins. The fraction that is not or is just loosely bound to macromolecules is termed free zinc and is involved in signal transduction [9]. Free zinc functions as a second messenger, regulating signal transduction cascades, for example, by inhibiting phosphatases and caspases [10,11]. Furthermore, zinc is closely connected to the cellular redox metabolism and redox signaling pathways [12], and the dysregulation of zinc homeostasis or redox metabolism is often associated with diseases, such as cancer or autoimmune diseases [13]. In breast cancer, free zinc and dysregulated zinc homeostasis are involved in crucial processes, such as epithelial-mesenchymal transition (EMT) and cytostatic resistance [14,15].

Investigating zinc homeostasis in mammary cells is essential for understanding the molecular regulation of cell growth and survival, which might improve treatment options for breast cancer and, thereby, patient health. At present, several groups have already monitored the changes in free zinc in different breast cancer cell lines using fluorescent probes [14,16], but the actual free zinc concentration in these cells is rarely reported. Moreover, the use of different probes hinders comparisons between the studies. Consequently, the present study compares the suitability of three commonly used low-molecular-weight fluorescent probes for determining the free zinc concentrations in different mammary cell lines in order to provide improved means for the investigation of free zinc in breast cancer.

## 2. Materials and Methods

### 2.1. Materials

Bovine serum albumin (BSA) (Sigma Aldrich, Munich, Germany), phosphate-buffered saline (PBS) (PAN-Biotech, Aidenbach, Germany), Dulbecco’s modified Eagle medium (DMEM) (PAN-Biotech, Aidenbach, Germany), fetal calf serum (FCS) (PAN-Biotech, Aidenbach, Germany), FluoZin-3 AM ester (FZ3) (Thermo Fisher Scientific, Waltham, MA, USA), mammary epithelial cell growth medium (MEGM) (Merck, Taufkirchen, Germany), *N*,*N*,*N*′,*N*′-Tetrakis(2-pyridylmethyl)ethylenediamine (TPEN) (Sigma Aldrich, Munich, Germany), 6-methoxy-8-*p*-toluenesulfonamido-quinoline (TSQ) (Enzo Biochem, Inc., Farmingdale, NY, USA), ZinPyr-1 (ZP1) (Santa Cruz Biotechnology, Dallas, TX, USA), and ZnSO_4_·7H_2_O (Sigma-Aldrich, Steinheim, Germany) were used in this study. All other chemicals were of analytical purity and purchased from standard sources.

### 2.2. Cell Culture

Four different cell lines representing different types of mammary cells (Table 1) were used in this study. The cell lines T47D [17] and MDA-MB-231 [18] were purchased from the DSMZ—German Collection of Microorganisms and Cell Cultures GmbH (Braunschweig, Germany). The cell lines MCF10A [19] and MCF7 [20] are available from the American Type Culture Collection (ATCC; Manassas, VA, USA). The MCF7, T47D, and MDA-MB-231 cells were cultured at 37 °C with 5% CO_2_ in a humidified atmosphere in Dulbecco’s modified Eagle medium (DMEM) without phenol red and containing 10% fetal calf serum (FCS), 100 U/mL penicillin, and 100 µg/mL streptomycin. The MCF10A cells were cultured in mammary epithelial cell growth medium (MEGM), consisting of mammary epithelial cell basal medium, 10% FCS, 100 U/mL penicillin, 100 µg/mL streptomycin, 100 ng/mL cholera toxin, and mammary epithelial cell growth medium supplements (bovine pituitary extract: 0.004 mL/mL, epidermal growth factor (recombinant; human): 10 ng/mL, insulin (recombinant; human): 5 µg/mL, and hydrocortisone: 0.5 µg/mL).

### 2.3. Dye Loading and Fluorescence Measurements

The low-molecular-weight fluorescent probes ZinPyr-1 (ZP1) [23], 6-methoxy-8-*p*-toluenesulfonamido-quinoline (TSQ) [24], and FluoZin-3 AM (FZ3) [25] were used to monitor intracellular free zinc. The cells were seeded in 96-well plates in the respective growth media for 48 h (MCF7: 50,000 cells/cavity; MDA-MB-231: 50,000 cells/cavity; T47D: 80,000 cells/cavity; MCF10A: 20,000 cells/cavity). After washing with assay buffer (120 mM NaCl, 5.4 mM KCl, 5 mM glucose, 1 mM CaCl_2_, 1 mM MgCl_2_, 1 mM NaH_2_PO_4_, and 10 mM HEPES; pH 7.35), the cells were loaded for 30 min with typical concentrations of ZP1 (2.5 µM) [26], TSQ (100 µM) [27], or FZ3 (0.75 µM) [28], respectively, all diluted in assay buffer containing 0.3% bovine serum albumin (BSA). After washing twice with the assay buffer, the measurements were performed in either 100 µL of growth medium or assay buffer. To determine the minimal (F_Min_) and maximal (F_Max_) fluorescence, a subset of wells was incubated with either 200 µM of the chelator *N*,*N*,*N*′,*N*′-Tetrakis(2-pyridylmethyl)ethylenediamine (TPEN) or 1 mM ZnSO_4_, respectively. After incubation for 20 min at 37 °C, the baseline fluorescence was measured (ZP1: λ_ex_ = 508 nm, λ_em_ = 527 nm; FZ3: λ_ex_ = 497 nm, λ_em_ = 519 nm; TSQ: λ_ex_ = 368 nm, λ_em_ = 482 nm) with a fluorescence plate reader (SPARK; Tecan, Switzerland) for 10 min in 2 min intervals. Subsequently, 20 µL of 6-fold concentrated ZnSO_4_ was added to determine the zinc uptake for further 30 min. In the cavities for F_Min_ and F_Max_, 20 µL of TPEN or ZnSO_4_ solutions, respectively, was added to compensate for potential volume effects. The fractional saturation was calculated as follows: [Fractional saturation] = [(F − F_Min_)/(F_Max_ − F_Min_)]. The free zinc calculation was based on the equation by Grynkiewicz et al., [Zn_free_] = (K_D_) × [(F − F_Min_)/(F_Max_ − F)] [29], using the dissociation constants (K_D_) for the zinc/probe complexes of 8.9 nM for FZ3 [30] and 0.7 nM for ZP1 [23], respectively.

### 2.4. Confocal Laser Scanning Microscopy

For the cell imaging, the cells (MCF7: 25,000 cells/cavity; MDA-MB-231: 19,000 cells/cavity; T47D: 38,000 cells/cavity; MCF10A: 6000 cells/cavity) were grown on µ-Slide 8-well plates (ibidi; Gräfelfing, Germany). After 24 h, the medium was renewed, and the cells were incubated in their respective growth media for 72 h. As described above, the cells were loaded with ZP1 in assay buffer with 0.3% BSA for 30 min. After washing the cells twice, the medium was added, and images were taken with a Leica TCS SP8 CLMS equipped with an LAS X 3.5.5.19976 software platform (Leica, Wetzlar, Germany) using an HC PL APO CS2 63×/1.20 water objective. For the excitation, a 488 nm laser was used. The detector was set to a bandwidth of 500–550 nm.

### 2.5. Statistical Analysis

The data are shown as the means of at least three independent experiments. The statistical analyses were performed with the GraphPad Prism software, version 8.02 (GraphPad Software Inc., San Diego, CA, USA). The normal distributions of the data sets were tested using the Anderson–Darling test. The statistical significance of the experimental results was calculated using a one-way analysis of variance (ANOVA) with Holm–Sidak’s multiple comparison test or a two-way analysis of variance (ANOVA) with Tukey´s or Dunnett´s multiple comparison tests, as indicated in the respective figure legends.

## 3. Results

### 3.1. Characterization of FZ3 for the Detection of Free Intracellular Zinc

**Figure 1:**
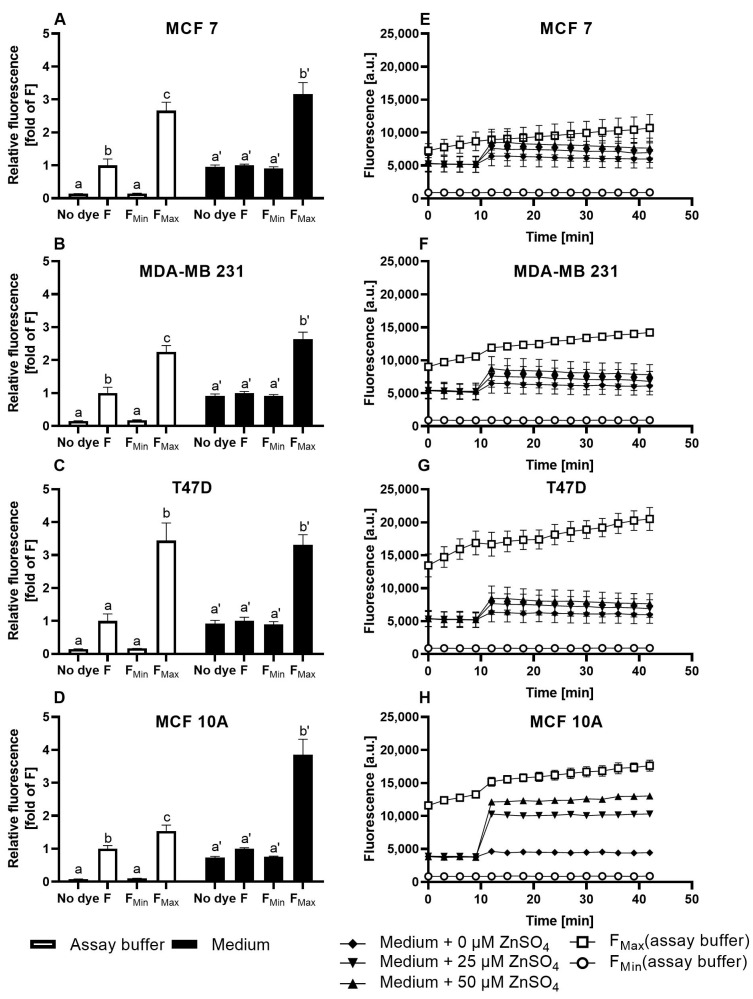
Cellular FZ3 fluorescence in untreated cells and in response to zinc incubation. The MCF7 (**A**,**E**), MDA-MB-231 (**B**,**F**), T47D (**C**,**G**), and MCF10A (**D**,**H**) cells were either left untreated (no dye) or loaded with 0.75 µM FZ3. To induce F_Min_ and F_Max_, the cells were pre-incubated for 20 min with final concentrations of 200 µM TPEN or 1 mM ZnSO_4_, respectively. For the zinc uptake experiments, the indicated amounts of ZnSO_4_ were added after the baseline measurements in the control medium for 10 min, and the fluorescence was monitored for an additional 30 min. The measurements were performed in either assay buffer (white bars/symbols) or cell culture medium (black bars/symbols) and are shown at the end of the experiment normalized to F (**A**–**D**) or as kinetics of the arbitrary fluorescence units (**E**–**H**). All data are shown as the means + SEM of at least *n* = 3 independent experiments. For (**A**–**D**), the significant differences between the different parameters (no dye, F, F_Min_, and F_Max_) are indicated by bars not sharing the same plain letter (a, b, c) for the assay buffer or a letter with an inverted comma (a′, b′) for the medium (a one-way analysis of variance (ANOVA) with Holm–Sidak´s multiple comparison test).

The suitability of FZ3 for the detection of free intracellular zinc in mammary cells is challenged by the data shown in Figure 1. The data show that the fluorescence in the medium differs significantly from the observations in the assay buffer, indicating that the surrounding liquid that is added after the dye loading has a profound impact on the results (Figure 1A–D). During the measurements in the assay buffer, which is a simplistic osmo-ion-balanced buffer, the basal fluorescence of the FZ3-loaded cells (F) is reduced when incubated with the chelator TPEN, generating a minimal FZ3-dependent fluorescence signal (F_Min_), which is of comparable intensity to the cellular autofluorescence (w/o dye loading). In contrast, in the cell culture medium, there is no statistically significant difference between the F, F_Min_, and the cellular autofluorescence (Figure 1A–D; black bars). In both the assay buffer and culture medium, the addition of 1 mM ZnSO_4_ induces a pronounced increase in the FZ3-dependent signal in all four cell lines, representing saturation with zinc (F_Max_). Yet, the relative increase in the fluorescence is markedly different in the MCF10A cells, depending on the surrounding medium (Figure 1A–D).

In the assay buffer, the basal free zinc concentrations in the four investigated cell lines range between 3.0 nM and 22.3 nM (MCF7: 4.6 ± 1.5 nM; MDA-MB-231: 5.9 ± 1.6 nM; T47D: 3 ± 0.5 nM; MCF10A: 22.3 ± 14.8 nM). In contrast, the experimental setup based on the culture medium does not result in the detection of significant amounts of basal free intracellular zinc.

**Figure 2:**
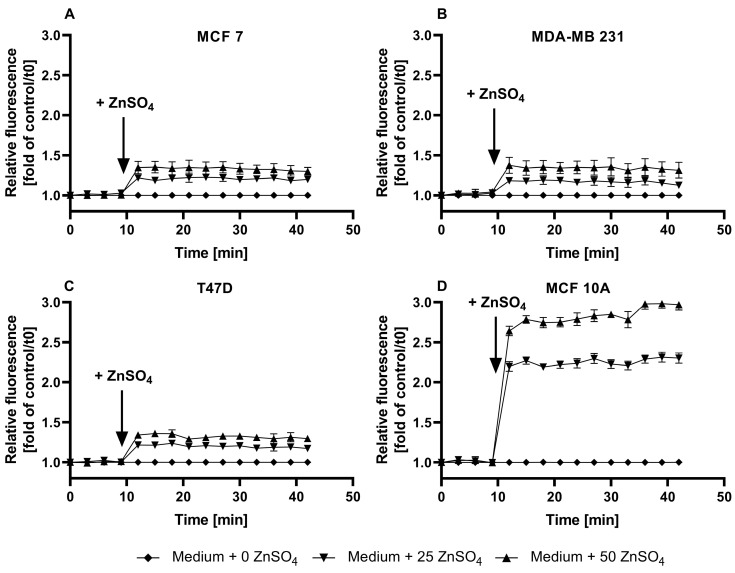
Zinc uptake into MCF7, MDA-MB-231, T47D, and MCF10A cells measured by FZ3. The MCF7 (**A**), MDA-MB-231 (**B**), T47D (**C**), and MCF10A (**D**) cells were either left untreated (no dye) or loaded with 0.75 µM FZ3. The data from Figure 1E–H are shown as the time-dependent fluorescence normalized to the control at t = 0 min. The data are shown as the means + SEM of at least three independent experiments. The significant differences between the different zinc incubations and the control at the respective times are indicated in the supplementary materials (Supplementary Table S1).

In the time-resolved measurements of the zinc uptake, the addition of ZnSO_4_ to the FZ3-loaded cells results in a swift increase in the fluorescence in the first two minutes, followed by a plateau. While the impact of zinc is moderate in the MCF7, MDA-MB-231, and T47D cells (Figure 1E-G), the MCF10A cells respond with a stronger elevation in the fluorescence signal (Figure 1H). Normalized to baseline fluorescence (Figure 2), the addition of extracellular ZnSO_4_ results in an increase of approximately 1.2-fold (for 25 µM) and 1.4-fold (for 50 µM) for the basal FZ3-accessible zinc in the MCF7, MDA-MB-231, and T47D cells. In the MCF10A cells, a stronger fluorescence elevation results in values up to three times the basal fluorescence intensity (Figure 2D).

Taken together, FZ3 is suited to indicate the zinc uptake in mammary cells. In contrast, it is not suitable for the reliable quantification of free zinc concentrations under typical cell culture conditions due to the significant differences in basal fluorescence (F) and cellular autofluorescence, depending on the surrounding medium, resulting in a lack of quantifiable free intracellular zinc during the measurements in the culture medium.

### 3.2. Evaluation of TSQ for the Measurement of Free Intracellular Zinc

**Figure 3:**
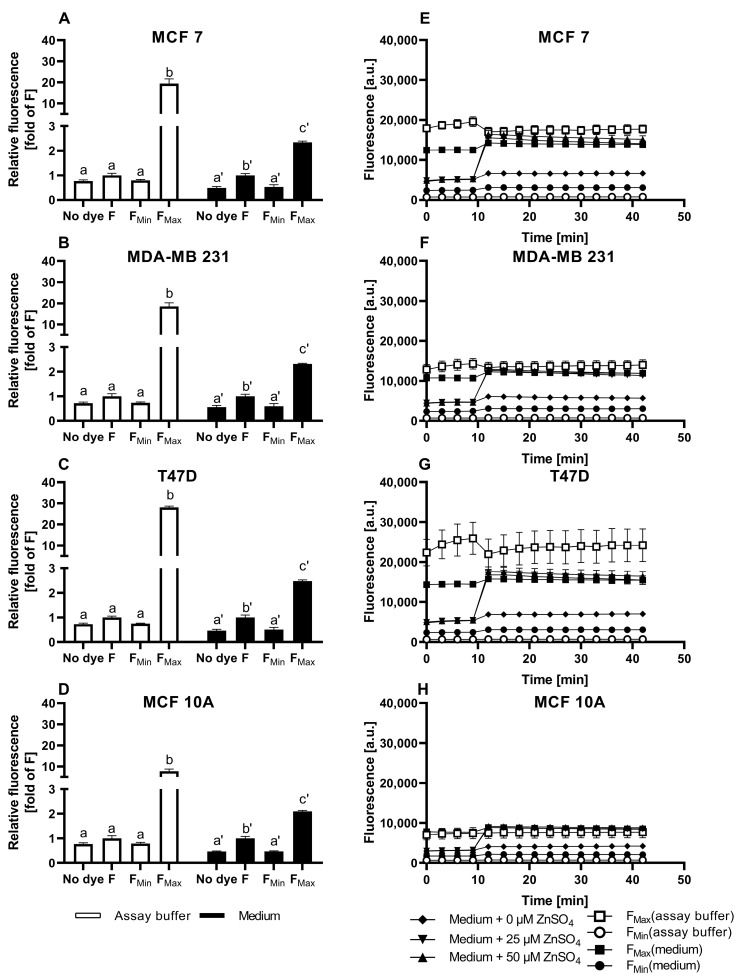
Cellular TSQ fluorescence in untreated cells and in response to zinc incubation. The MCF7 (**A**,**E**), MDA-MB-231 (**B**,**F**), T47D (**C**,**G**), and MCF10A (**D**,**H**) cells were either left untreated (no dye) or loaded with 100 µM TSQ. To induce F_Min_ and F_Max_, the cells were pre-incubated for 20 min with final concentrations of 200 µM TPEN or 1 mM ZnSO4, respectively. For the zinc uptake experiments, the indicated amounts of ZnSO_4_ were added after the baseline measurements in the control medium for 10 min, and the fluorescence was monitored for an additional 30 min. The measurements were performed in either assay buffer (white bars/symbols) or cell culture medium (black bars/symbols) and are shown at the end of the experiment normalized to F (**A**–**D**) or as the kinetics of the arbitrary fluorescence units (**E**–**H**). All of the data are shown as the means + SEM of at least *n* = 3 independent experiments. For (**A**–**D**), the significant differences between the different parameters (no dye, F, F_Min_, and F_Max_) are indicated by bars not sharing the same plain letter (a, b) for the assay buffer or a letter with an inverted comma (a′, b′, c′) for the medium (a one-way analysis of variance (ANOVA) with Holm–Sidak´s multiple comparison test).

In the TSQ-loaded cells, a clear distinction between the basal cellular TSQ fluorescence F, F_Min_, and the cellular autofluorescence is only observed in the culture media but not in the assay buffer (Figure 3A–D). The addition of 1 mM ZnSO_4_ for saturation of the sensor (F_Max_) leads to a significant increase in the fluorescence in all of the experiments. However, the relative increase from F to F_Max_ is visibly lower in the media compared to the assay buffer, resulting in higher values for F_max_ when the data are normalized to F. The calculated fractional saturation of TSQ in the mammary cell lines in the assay buffer is in the range of 0.01 to 0.03. (MCF7: 0.011 ± 0.002; MDA-MB-231: 0.015 ± 0.005; T47D: 0.010 ± 0.002; MCF10A: 0.030 ± 0.007). In the medium, on the other hand, the fractional saturation of TSQ is in the range of 0.23 to 0.33. (MCF7: 0.26 ± 0.02; MDA-MB-231: 0.23 ± 0.01; T47D: 0.26 ± 0.04; MCF10A: 0.33 ± 0.04). The calculation of free zinc is hindered by the formation of two different kinds of complexes, Zn(TSQ)_2_ as well as ternary complexes of TSQ with protein-bound zinc [31].

**Figure 4:**
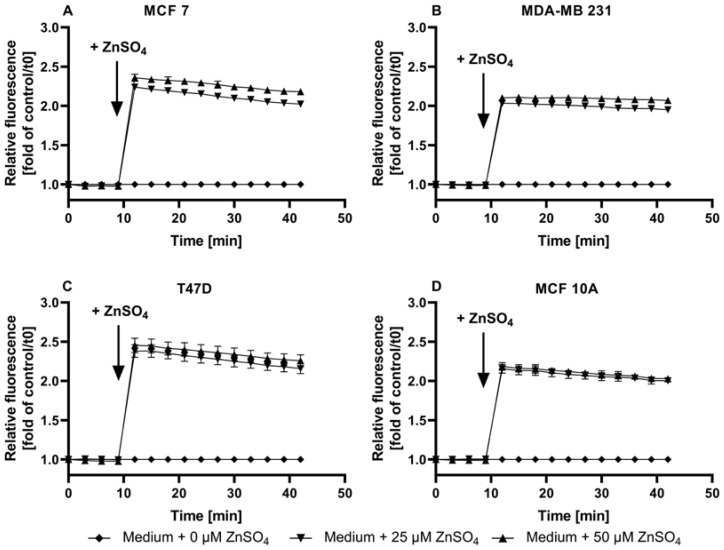
Zinc uptake into MCF7, MDA-MB-231, T47D, and MCF10A cells, measured by TSQ. The MCF7 (**A**), MDA-MB-231 (**B**), T47D (**C**), and MCF10A (**D**) cells were either left untreated (no dye) or loaded with 100 µM TSQ. The data from Figure 3E–H are shown as the time-dependent fluorescence normalized to the control at t = 0 min. The data are shown as the means + SEM of at least three independent experiments. The significant differences between the different zinc incubations and the control at the respective times are indicated in the supplementary materials (Supplementary Table S2).

In all of the cell lines, the addition of ZnSO_4_ increases the fluorescence of TSQ without dose dependency. A zinc concentration of 25 µM is already sufficient to saturate the intracellular amount of TSQ, with a resulting fluorescence comparable to F_Max_ (Figure 3E–H). This saturation is also reflected in the normalized plot of the TSQ fluorescence after the Zn addition (Figure 4A–D), resulting in an approximate doubling of the fluorescence intensity for all of the cell lines.

Taken together, TSQ does detect free zinc in mammary cells, but its use is hindered by the following issues: (i) visible differences between the assay buffer and the medium with regard to F_Max_; (ii) no statistically significant difference between F, F_Min_, and the cellular autofluorescence in the assay buffer; (iii) dye saturation by the addition of low amounts of zinc, indicating an insufficient dynamic range; (iv) no feasibility of determining the free zinc concentrations.

### 3.3. Application of ZP1 for Detection and Quantification of Intracellular Free Zinc

**Figure 5:**
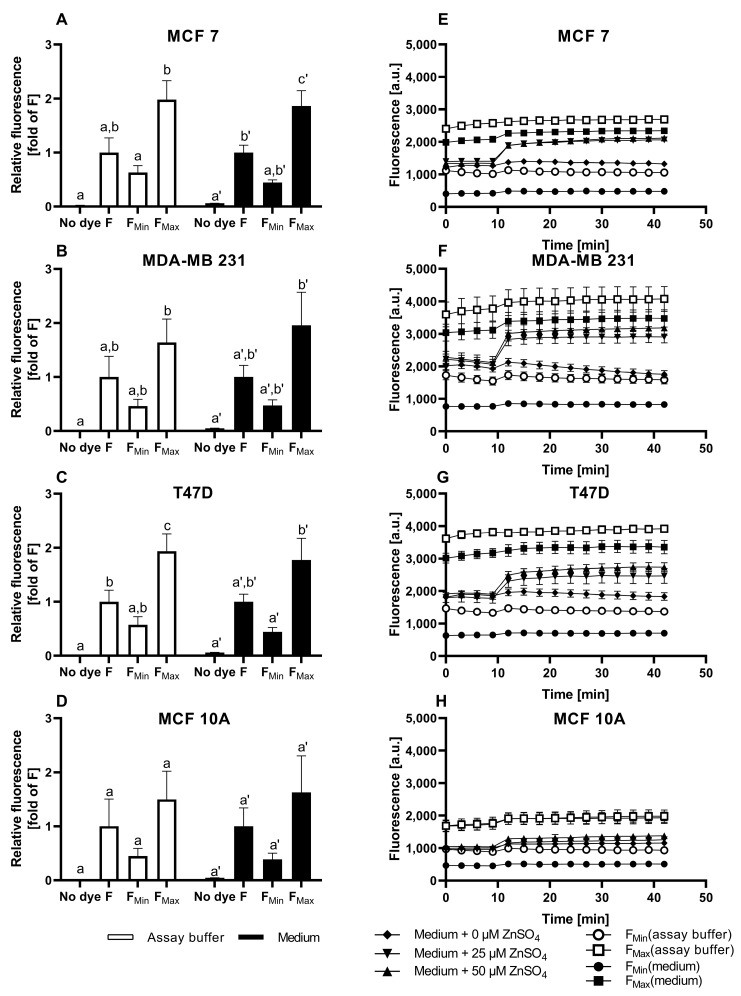
Cellular ZP1 fluorescence in the untreated cells and in response to zinc incubation. The MCF7 (**A**,**E**), MDA-MB-231 (**B**,**F**), T47D (**C**,**G**), and MCF10A (**D**,**H**) cells were either left untreated (no dye) or loaded with 2.5 µM ZP1. To induce F_Min_ and F_Max_, the cells were pre-incubated for 20 min with final concentrations of 200 µM TPEN or 1 mM ZnSO_4_, respectively. For the zinc uptake experiments, the indicated amounts of ZnSO_4_ were added after the baseline measurements in the control medium for 10 min, and the fluorescence was monitored for an additional 30 min. The measurements were performed in either assay buffer (white bars/symbols) or cell culture medium (black bars/symbols) and are shown at the end of the experiment normalized to F (**A**–**D**) or as the kinetics of the arbitrary fluorescence units (**E**–**H**). All of the data are shown as the means + SEM of at least *n* = 3 independent experiments. For (**A**–**D**), the significant differences between the different parameters (no dye, F, F_Min_, and F_Max_) are indicated by bars not sharing the same plain letter (a, b, c) for the assay buffer or a letter with an inverted comma (a′, b′, c′) for the medium (a one-way analysis of variance (ANOVA) with Holm–Sidak´s multiple comparison test).

**Figure 6:**
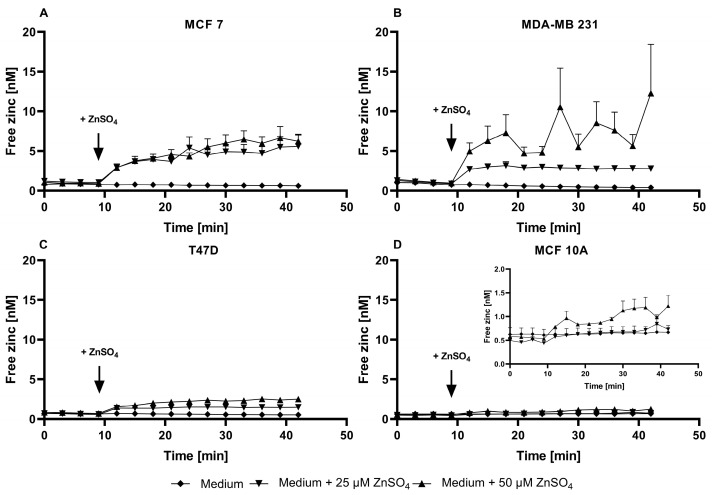
Zinc uptake into MCF7, MDA-MB-231, T47D, and MCF10A cells measured by ZP1. The MCF7 (**A**), MDA-MB-231 (**B**), T47D (**C**), and MCF10A (**D**) cells were either left untreated (no dye) or loaded with 2.5 µM ZP1. The data from Figure 3E–H are shown as the time-dependent fluorescence normalized to the control at t = 0 min. The data are shown as the means + SEM of at least three independent experiments. The significant differences between the different zinc incubations and the control at the respective times are indicated in the supplementary materials (Supplementary Table S3).

**Figure 7:**
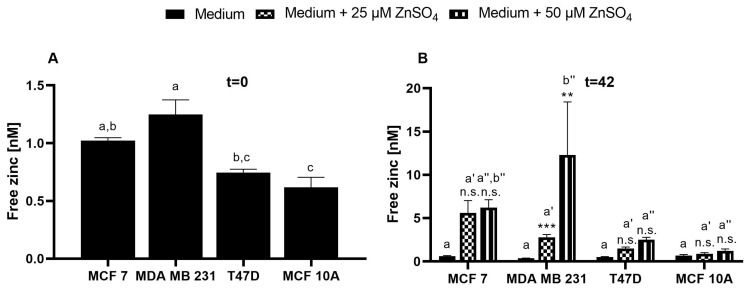
Free zinc concentrations in MCF7, MDA-MB-231, T47D, and MCF10A cells detected by ZP1. The cells were treated as described in the legend of Figure 5, and the cellular free zinc concentrations in the resting cells (t = 0 min) (**A**) or after the zinc incubation (t = 42 min) (**B**) were calculated. The data are shown as the means + SEM of four independent experiments. (**A**) The bars sharing the same letter are not significantly different (a one-way analysis of variance (ANOVA) with Holm–Sidak´s multiple comparison test). (**B**) Within each cell line, the significant differences from the control are indicated by asterisks (** *p* < 0.002; *** *p* < 0.001; n.s., not significant). The significant differences between the cell lines at the same zinc concentrations are indicated by different letters (a for controls; a′ for 25 µM ZnSO_4_; a″, b″ for 50 µM ZnSO_4_) (a two-way analysis of variance (ANOVA) with Tukey´s multiple comparison test).

After loading with ZP1, the fluorescence parameters were comparable in the assay buffer and culture medium (Figure 5A–D). The cells show negligible autofluorescence using the ZP1 excitation/emission wavelengths of 492/527 nm. Furthermore, the addition of TPEN leads to the F_Min_ values exceeding those in the absence of the dye, indicating noteworthy autofluorescence of the probe in its zinc-free state. The addition of zinc results in a concentration-dependent elevation in the fluorescence that remains within the limits of the F_Min_ and F_Max_ values (Figure 5E–H), allowing for the calculation of free Zn (Figure 6 and Figure 7). ZP1 shows a bi-phasic trend for the zinc uptake, in which there is a fast increase in the free zinc concentration in the first two minutes, followed by a moderate further increase during the subsequent observation period of 30 min (Figure 6).

The baseline free zinc concentrations vary between the four cell lines, with the MCF10A and MDA-MB-231 cells having the lowest and highest basal free zinc concentrations, respectively (Figure 7A). Moreover, by the end of the observation period, the free zinc concentration increases significantly and dose-dependently after the addition of zinc sulfate in all of the cell lines except for the MCF10A cells (Figure 7B). Even the addition of 50 µM ZnSO_4_ leads only to a slightly higher level of free intracellular zinc of approximately 1 nM in the MFC10A cells, which does not differ with statistical significance from the untreated control (Figure 7B). For the remaining cells, ZP1 detects a concentration-dependent uptake of zinc in the order of T47D < MCF7 < MDA-MB-231, with the latter showing the highest cellular free zinc levels of approximately 12 nM after an incubation with 50 µM extracellular Zn (Figure 7B).

**Figure 8:**
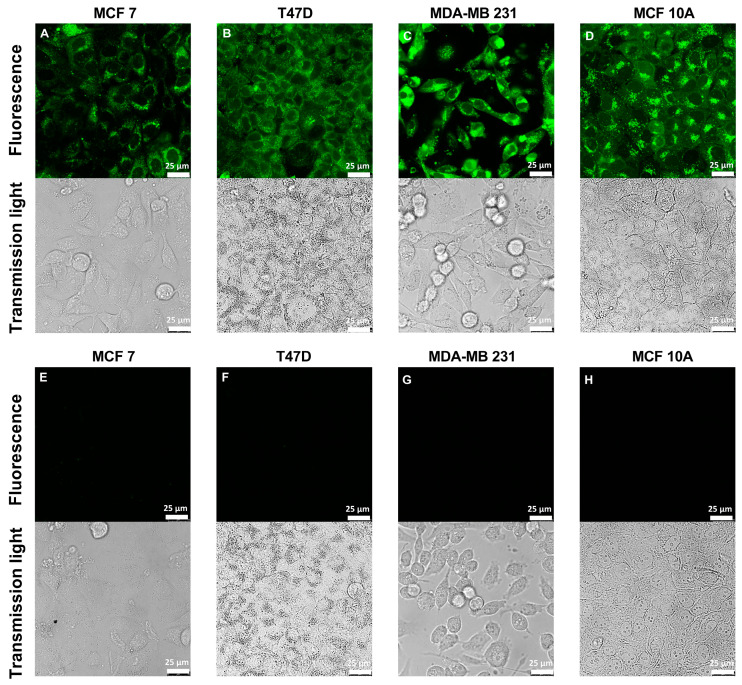
Intracellular distribution of ZP1-accessible zinc in MCF7, MDA-MB-231, T47D, and MCF10A cells. The cells were grown in culture medium for 96 h, either loaded with 2.5 µM ZP1 (**A**–**D**) or left untreated (**E**–**H**), and investigated by confocal laser scanning microscopy. The scale bar is 25 µm. The representative images of *n* = 3 independent experiments are shown.

In summary, ZP1 seems to be a more suitable probe for free Zn quantification in mammary cells in comparison with FZ3 and TSQ. ZP1 yields similar results in the assay buffer and culture medium, providing a fractional saturation sufficient for the calculation of free zinc and detecting zinc uptake. Notably, the MCF10A cells show the weakest uptake with ZP1, while the opposite is seen for FZ3. This highlights the relevance of considering the zinc pool(s) detected by the respective probe. For ZP1, the fluorescence imaging indicates the distribution of ZP1-accessible zinc throughout the cells, mostly in the cytosol and cytoplasmic structures, indicating the accumulation of substantial amounts of free zinc within the subcellular compartments (Figure 8).

## 4. Discussion

Free zinc is indispensable for many cellular processes with relevance for cancer cell growth and survival, such as signal transduction, proliferation, or apoptosis, and altered free zinc concentrations or a dysregulated zinc homeostasis are associated with several diseases, including cancer [15,32]. In breast cancer, the dysregulation of zinc homeostasis has been described on various levels, such as the zinc transporter network, total zinc content, or spatial distribution [5,6]. Since free zinc regulates some essential signaling processes, including the inhibition of phosphatases, the free zinc concentration and its fluctuations are a promising subject for investigation. This is particularly relevant in light of the remarkable role of the LIV-1 family of zinc transporters in breast cancer and the regulation of free zinc signaling by casein kinase 2, which have been extensively investigated by the group of Kathryn Taylor [33].

Low-molecular-weight fluorescent sensors are a central tool for the investigation of intracellular free zinc and have been widely used to determine the free zinc levels in multiple cell types [34,35], such as immune [36] or intestinal cells [37]. So far, in malignant or non-malignant mammary cells, they have been mainly deployed for evaluating the relative changes of free zinc or the visualization of zinc distribution. In contrast, calculations of the free zinc concentration in breast cancer cell lines have rarely been performed in vitro, and, to the best of our knowledge, they have not yet been performed in vivo [16,38,39,40,41]. Generally, apart from studies on free zinc, fluorescent low-molecular-weight molecules are an important tool in both in vitro and in vivo studies for many research fields, such as the detection of organophosphates or the investigation of kidney diseases [42,43,44].

This work compares three frequently applied low-molecular-weight fluorescent probes with respect to their suitability for the quantification of free zinc levels in three breast cancer cell lines (MCF7, T47D, and MDA-MB-231) and the non-malignant cell line MCF10A. Using FZ3, the relative changes in Zn-dependent fluorescence can be detected in culture media, but free zinc quantification is hindered by the lack of a significant difference between F and F_Min_, which would be required for sensor calibration (Figure 1A–D). Despite that, FZ3 can be used to determine the cellular zinc status or zinc uptake in cell culture medium by depicting the time-dependent fluorescence intensity (Figure 1E–H) [14,16,38]. The basal fluorescence levels differ between the cell lines. For example, the basal fluorescence of the MCF10A cells (Figure 1H) is lower than that of the other cell lines (Figure 1E–G). For comparison of the FZ3-based measurement of the zinc status or uptake between the different cell lines (Figure 2A–D), normalization of the fluorescence units is recommended, as described for FZ3 in HC11 mouse mammary cells [45]. Still, the normalized values are only poorly comparable to other studies.

The free zinc concentration can be determined with FZ3 in an extracellular osmo-ion-balanced assay buffer. This yields an order of magnitude suitable for comparison with other results or for estimating the impact on signaling processes, e.g., by considering it in relation to the IC_50_ values for phosphatases [10]. However, the differences in the data from the cell culture medium are remarkable, and it has to be questioned to what extent an assay buffer that starves the cells of their required growth factors represents a suitable environment for investigating the cellular free zinc status or uptake with relevance for cancer cells in a living organism. Fetal calf serum or MEGM supplements added to cell culture media introduce a variety of components that are important for proper cell growth, survival, or proliferation. Yet, they also affect the results, in particular, by contributing to extracellular metal ion buffering through binding to serum proteins, first and foremost albumin, thereby impacting the zinc uptake and cellular zinc homeostasis [9,26,46].

TSQ was previously used for the qualitative investigation of zinc in kidney or colon cancer cell lines, for example, for the observation of cellular zinc distribution and uptake by fluorescence microscopy or for the calculation of the fractional saturation or fluorescence intensities [27,31]. Yet, TSQ does not solely detect free zinc, as it can also associate with protein-bound zinc, resulting in ternary TSQ-Zn-protein complexes [31]. In Figure 4A–D, the qualitative zinc uptake into the mammary cells seemed to be already saturated in response to 25 µM of extracellular Zn, approximately doubling the fluorescence in all of the four cell lines. These observations with TSQ highlight the usefulness of calibrations, even in cases where absolute concentration is not required. The measured values approached F_Max_, illustrating a limited capacity to detect elevations in free zinc that might otherwise go unnoticed. Such an inadequate dynamic range could be compensated by changing the dye loading conditions, especially the amount of probe, but as the cells were already loaded with 100 µM, a substantial further increase seems impractical. It is well documented that the intracellular sensor concentration and the K_D_ value of the sensor influence the equilibrium between free and protein-bound zinc, and high probe concentrations can interfere with the cellular zinc homeostasis [30,47].

ZP1 had not previously been used for subtype-specific assessments of intracellular zinc pools or zinc responses in breast cancer. It detected differences in the basal free zinc levels among the cell lines, ranging between 0.62 nM and 1.25 nM (MCF7: 1.02 ± 0.04 nM; MDA-MB-231: 1.25 ± 0.22 nM; T47D: 0.74 ± 0.05 nM; MCF10A: 0.62 ± 0.15 nM). This is in agreement with the intracellular free zinc concentrations typically reported for in vitro studies to be picomolar to low nanomolar [48]. Comparable to our approach, there are a few studies in the literature that have also calculated the free zinc concentrations in mammary cell lines, but none of them compares different subtypes [49,50,51]. Hwang et al. reported a free zinc concentration in assay buffer-incubated MCF7 cells of 0.066 nM using FZ3 [49], which is approximately 100-fold lower compared to the 4.6 nM derived for FZ3 from Figure 1 and still one order of magnitude below the results with ZP1. This may be because of several reasons, including the use of various experimental approaches for the determination of the fluorescence intensities used to calculate the free zinc concentrations. Among them, one major factor is the choice of different methods for detecting fluorescence, which can be based on a microplate reader [28,49], a fluorescence microscope [51], or a flow cytometer [36,41], each of which has a substantial influence on the results [28]. For breast cancer cells, this choice will mainly be microscopy or plate readers, whereas flow cytometry is only of very limited relevance as the cells grow adherent and the required detachment might interfere with the results.

Other studies in breast cancer cell lines measured the free zinc levels in MCF7 and MCF10A cells using genetically encoded protein sensors that are targeting specific cellular compartments. In the MCF10A [50] and MCF7 [51] cells, the cytosolic concentrations of 0.08 nM and 0.44 nM were measured, respectively. In the endoplasmic reticulum (ER) of the MCF7 cells, the concentration of free zinc was found to be slightly higher (0.54 nM) than in the cytosol [51]. Taken together, our results for the MCF7 and MCF10A cells are comparable with those reported in the literature, while the free zinc concentrations for the MDA-MB-231 and T47D cells were not previously reported.

One notable difference between the fluorescent probes is observed in response to the addition of extracellular zinc. FZ3 detects the strongest increase in the MCF10A cells, whereas ZP1 shows the exact opposite. Most likely, both probes localize into different intracellular compartments, indicating different pools of free intracellular zinc. In contrast to TSQ, ZP1 and FZ3 are known for their rapid localization into cellular compartments [52,53]. While ZP1 is localized throughout the cells in the organelles and the cytosol in Figure 8, the literature reports for FZ3 indicate an exclusively vesicular localization of fluorescence in the MCF10A, T47D, and MDA-MB-231 cells [5]. Yet, this cannot be generalized because the FZ3 fluorescence was shown to be rather uniform with no punctuate staining pattern for the MCF7 cells [54]. In contrast to our result, the uptake of extracellular zinc resulted in a more pronounced increase in the FZ3 fluorescence for the MCF7 cells compared to the MCF10A cells in a previous study [55]. One notable difference was the investigation of ionophore-mediated zinc uptake in the latter study, once again highlighting the need to consider all of the experimental details when comparing these types of results.

In addition to low-molecular-weight fluorescent probes, intracellular free zinc can also be measured by genetically encoded protein sensors, such as eCALWY, eZinCh, or ZapCV2 [34,50,51]. These offer the advantage of targeted localization to sub-cellular compartments. For example, the protein sensors eCALWY-4 and eZinCh-2 are located in the cytosol, whereas ER-eCALWY-4 and ER-eZinCh-2 are ER-targeted sensors [51]. This opens the possibility for the specific determination of different intracellular free zinc pools, if required. On the other hand, low-molecular-weight probes typically have a larger dynamic range and can be used after a relatively brief (30 min) and uncomplicated loading process. To combine the control of intracellular localization with the advantages of low-molecular-weight sensors, hybrid sensors are a promising topic of current research [56,57], which could become a valuable future tool for biomarker imaging in breast cancer [58].

## 5. Conclusions

The determination of free intracellular zinc in mammary cells could help in understanding the role of zinc homeostasis in breast cancer, thereby identifying better diagnostic and therapeutic options. While several studies did measure free zinc with low-molecular-weight fluorescent probes, these data currently have poor comparability, mainly due to the choice of different fluorescent probes, and only a few of them used calibrations and reported actual concentrations. Our results emphasize the potential impact of various factors, including the choice of probe, the environment in which the cells are kept during the measurements, the controls used to detect potential saturation, and the intracellular localization of the probe. Among the three commonly used probes compared in the present study, ZP1 seems most suited for the quantification of free intracellular zinc in mammary cells.

## Figures and Tables

**Table 1:** Classification of the cell lines used in the present study [21,22].

Cell Line	Primary Tumor/Cell Type	Receptor Expression			Classification
		Estrogen	Progesterone	HER2 Amplified	
MCF7	Invasive ductal carcinoma	+	+	−	Luminal A
T47D	Invasive ductal carcinoma	+	+	−	Luminal A
MDA-MB-231	Adenocarcinoma	−	−	−	Basal
MCF10A	Mammary epithelial cells	−	−	−	Non-tumor mammary cells

## Data Availability

The data presented in this study are available upon reasonable request from the corresponding author.

## Supplementary Materials

The following supporting information can be downloaded at https://www.mdpi.com/article/10.3390/nu15081873/s1, Table S1: *p* Values of significance test (two-Way ANOVA with Dunnett´s multiple comparisons test) for zinc uptake measured with FZ3 (Figure 2); Table S2: *p* Values of significance test (two-Way ANOVA with Dunnett´s multiple comparisons test) for zinc uptake measured with TSQ (Figure 4); Table S3: *p* Values of significance test (two-Way ANOVA with Dunnett´s multiple comparisons test) for zinc uptake measured with ZP1 (Figure 6).
