# Supplementary material for: Comparison of Three Low-Molecular-Weight Fluorescent Probes for Measuring Free Zinc Levels in Cultured Mammary Cells
Source: Nutrients. 2023 Apr 13;15(8):1873. doi: 10.3390/nu15081873 (PMC10141224; doi:10.3390/nu15081873)
Supplement: Supplementary file 1 [file nutrients-15-01873-s001.zip › nutrients-2281437-supplementary.pdf]

Supplemental Material

Comparison of Three Low-Molecular-Weight Fluorescent Probes for Measuring Free Zinc Levels in Cultured Mammary Cells

Christopher Hübner [1,†], Claudia Keil [1,†], Anton Jürgensen [1], Lars Barthel[2] and Hajo Haase [1,*]

[1] Department of Food Chemistry and Toxicology, Institute of Food Technology and Food Chemistry, Technische Universität Berlin, Straße des 17. Juni 135, 10623 Berlin, Germany
[2] Department of Applied and Molecular Microbiology, Institute of Biotechnology, Technische Universität Berlin, Straße des 17. Juni 135, 10623 Berlin, Germany
* Correspondence: haase@tu-berlin.de; Tel.: +49-(0)-30-31472701; Fax: +49-(0)-30-31472823
† These authors contributed equally to this work.

Citation: Hübner, C.; Keil, C.; Jürgensen, A.; Barthel, L.; Haase, H. Comparison of Three Low-Molecular-Weight Fluorescent Probes for Measuring Free Zinc Levels in Cultured Mammary Cells. *Nutrients* **2023**, *15*, 1873. https://doi.org/10.3390/nu15081873

Academic Editor: Roberto Iacone

Received: 28 February 2023
Revised: 31 March 2023
Accepted: 11 April 2023
Published: 13 April 2023

Copyright: © 2023 by the authors. Submitted for possible open access publication under the terms and conditions of the Creative Commons Attribution (CC BY) license (https://creativecommons.org/licenses/by/4.0/).

Suppl.-Table S1: P Values of significance test (two-Way ANOVA with Dunnett´s multiple comparisons test) for zinc uptake measured with FZ3 (Figure 2).

t	MCF 7		MDA-MB 231		T47D		MCF 10A	
	significance [p value to control at time t]		significance [p value to control at time t]		significance [p value to control at time t]		significance [p value to control at time t]	
	25 µM $ZnSO_4$	50 µM $ZnSO_4$	25 µM $ZnSO_4$	50 µM $ZnSO_4$	25 µM $ZnSO_4$	50 µM $ZnSO_4$	25 µM $ZnSO_4$	50 µM $ZnSO_4$
**0**	>0.99	>0.99	>0.99	>0.99	>0.99	>0.99	>0.99	>0.99
**3**	0.92	>0.99	0.97	0.9	0.95	0.94	0.82	0.89
**6**	0.97	>0.99	0.99	0.91	0.66	0.96	0.94	0.87
**9**	0.84	>0.99	0.89	0.78	>0.99	0.94	>0.99	>0.99
**12**	<0.001	<0.001	0.03	<0.001	<0.001	<0.001	<0.001	<0.001
**15**	0.003	<0.001	0.04	<0.001	<0.001	<0.001	<0.001	<0.001
**18**	<0.001	<0.001	0.02	<0.001	<0.001	<0.001	<0.001	<0.001
**21**	<0.001	<0.001	0.03	<0.001	<0.001	<0.001	<0.001	<0.001
**24**	<0.001	<0.001	0.06	<0.001	<0.001	<0.001	<0.001	<0.001
**27**	<0.001	<0.001	0.03	<0.001	<0.001	<0.001	<0.001	<0.001
**30**	0.002	<0.001	0.05	<0.001	<0.001	<0.001	<0.001	<0.001
**33**	0.001	<0.001	0.07	<0.001	<0.001	<0.001	<0.001	<0.001
**36**	<0.001	<0.001	0.03	<0.001	<0.001	<0.001	<0.001	<0.001
**39**	0.004	<0.001	0.08	<0.001	<0.001	<0.001	<0.001	<0.001
**42**	0.002	<0.001	0.17	<0.001	<0.001	<0.001	<0.001	<0.001

Suppl.-Table S2: P Values of significance test (two-Way ANOVA with Dunnett´s multiple comparisons test) for zinc uptake measured with TSQ (Figure 4).

t	MCF 7		MDA-MB 231		T47D		MCF 10A	
	significance [p value to control at time t]		significance [p value to control at time t]		significance [p value to control at time t]		significance [p value to control at time t]	
	25 µM $ZnSO_4$	50 µM $ZnSO_4$	25 µM $ZnSO_4$	50 µM $ZnSO_4$	25 µM $ZnSO_4$	50 µM $ZnSO_4$	25 µM $ZnSO_4$	50 µM $ZnSO_4$
**0**	>0.99	>0.99	>0.99	>0.99	>0.99	>0.99	>0.99	>0.99
**3**	0.64	0.72	0.98	0.95	>0.99	0.97	>0.99	0.97
**6**	0.76	0.76	0.94	0.86	>0.99	0.94	0,99	0.96
**9**	0.62	0.68	0.93	0.89	>0.99	0.93	>0,99	0.94
**12**	<0.001	<0.001	<0.001	<0.001	<0.001	<0.001	<0.001	<0.001
**15**	<0.001	<0.001	<0.001	<0.001	<0.001	<0.001	<0.001	<0.001
**18**	<0.001	<0.001	<0.001	<0.001	<0.001	<0.001	<0.001	<0.001
**21**	<0.001	<0.001	<0.001	<0.001	<0.001	<0.001	<0.001	<0.001
**24**	<0.001	<0.001	<0.001	<0.001	<0.001	<0.001	<0.001	<0.001
**27**	<0.001	<0.001	<0.001	<0.001	<0.001	<0.001	<0.001	<0.001
**30**	<0.001	<0.001	<0.001	<0.001	<0.001	<0.001	<0.001	<0.001
**33**	<0.001	<0.001	<0.001	<0.001	<0.001	<0.001	<0.001	<0.001
**36**	<0.001	<0.001	<0.001	<0.001	<0.001	<0.001	<0.001	<0.001
**39**	<0.001	<0.001	<0.001	<0.001	<0.001	<0.001	<0.001	<0.001
**42**	<0.001	<0.001	<0.001	<0.001	<0.001	<0.001	<0.001	<0.001

Suppl.-Table S3: P Values of significance test (two-Way ANOVA with Dunnett´s multiple comparisons test) for zinc uptake measured with ZP1 (Figure 6).

t	MCF 7 significance [p value to control at time t]		MDA-MB 231 significance [p value to control at time t]		T47D significance [p value to control at time t]		MCF 10A significance [p value to control at time t]	
	25 µM $ZnSO_4$	50 µM $ZnSO_4$	25 µM $ZnSO_4$	50 µM $ZnSO_4$	25 µM $ZnSO_4$	50 µM $ZnSO_4$	25 µM $ZnSO_4$	50 µM $ZnSO_4$
**0**	0.86	0.94	0.99	0.97	>0.99	0.73	0.64	0.95
**3**	0.95	>0.99	>0.99	0.99	0.93	0.95	0.52	0.92
**6**	0.95	>0.99	>0.99	>0.99	0.89	0.96	0.7	0.86
**9**	0.95	>0.99	>0.99	>0.99	0.94	0.96	0.52	0.87
**12**	0.03	0.03	0.53	0.07	0.009	0.001	0.94	0.57
**15**	0.003	0.002	0.42	0.01	0.005	<0.001	>0.99	0.06
**18**	<0.001	0.001	0.36	0.002	0.003	<0.001	>0.99	0.4
**21**	0.002	<0.001	0.43	0.11	<0.001	<0.001	>0.99	0.36
**24**	<0.001	<0.001	0.38	0.1	<0.001	<0.001	>0.99	0.31
**27**	<0.001	<0.001	0.39	<0.001	<0.001	<0.001	>0.99	0.18
**30**	<0.001	<0.001	0.4	0.04	<0.001	<0.001	>0.99	0.007
**33**	<0.001	<0.001	0.42	<0.001	<0.001	<0.001	>0.99	0.003
**36**	<0.001	<0.001	0.39	<0.001	<0.001	<0.001	0.89	0.002
**39**	<0.001	<0.001	0.39	0.03	<0.001	<0.001	0.45	0.13
**42**	<0.001	<0.001	0.39	<0.001	<0.001	<0.001	0.91	0.001
